# Supplementary material for: Extreme rejuvenation and softening in a bulk metallic glass
Source: Nat Commun. 2018 Feb 8;9:560. doi: 10.1038/s41467-018-02943-4 (PMC5805766; doi:10.1038/s41467-018-02943-4)
Supplement: Supplementary file 1 — Supplementary Information [file 41467_2018_2943_MOESM1_ESM.pdf]

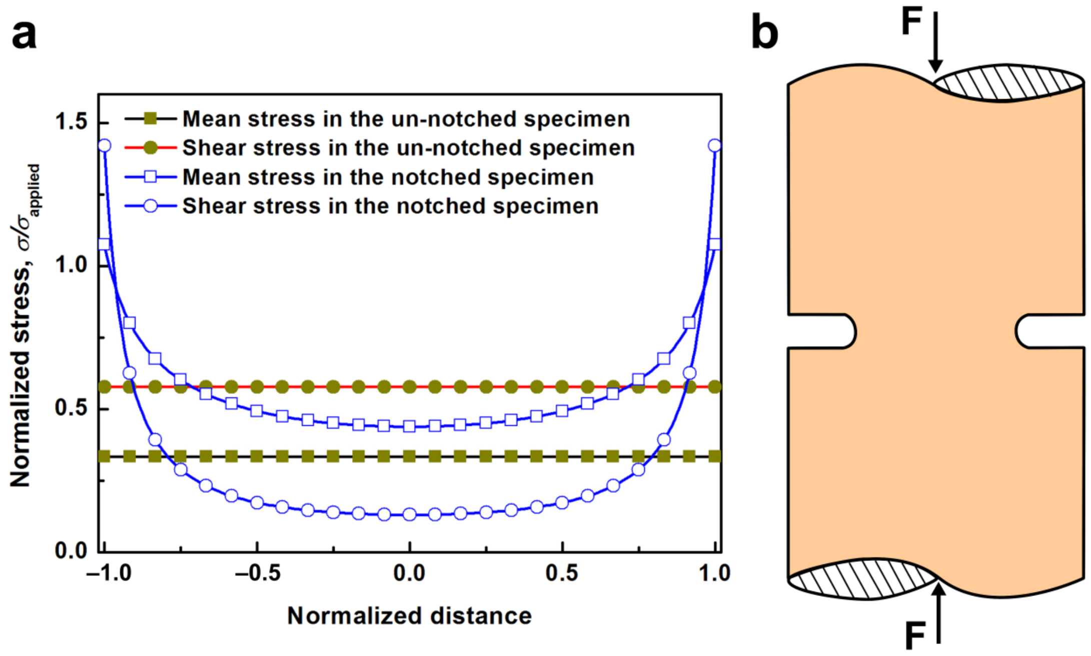

Supplementary Figure 1 │ Stress states in BMG specimens elastically loaded in axial compression. (a) Variation of mean stresses and shear stresses across a diameter in the central circular plane in notched and un-notched specimens. The stresses in the notched specimen are calculated based on Neuber's solution[1]. Compared to the un-notched specimen, the shear stress in the notch region is greatly reduced. This, and the constraint of the lower-stressed material outside the notch region, suppress shear-banding and favour homogeneous flow even at RT. The applied stress σ_{applied} is calculated for the full cylindrical cross-section of an un-notched specimen, and for the area of the disc defined by the notch in a notched specimen. **(b)** Schematic diagram of the notched specimen.

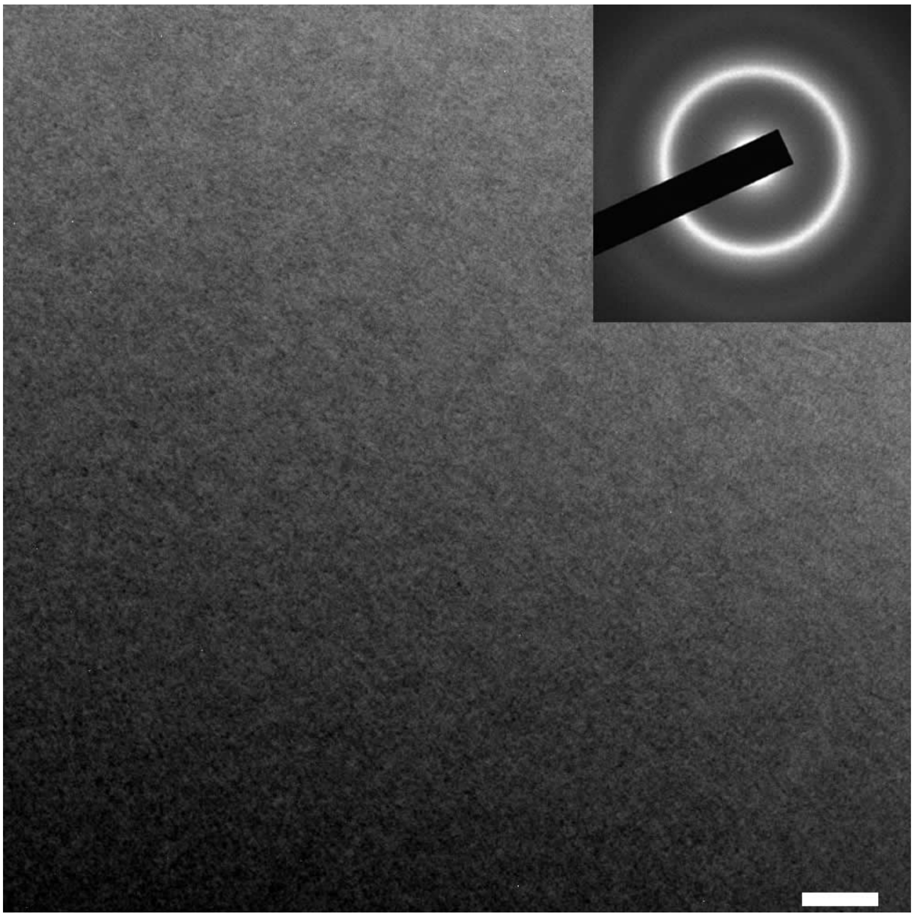

Supplementary Figure 2 │ Transmission electron microscopy (TEM) image and selected-area electron diffraction pattern from the centre of a deformed notched specimen. The sample is from the central region of the disc defined by the notch (see Supplementary Fig. 3). The disc was sectioned from a specimen that had been subjected to 40% compressive strain at RT (this is the axial strain within the disc itself, calculated from the reduction in the width of the notch). There is no trace of any crystalline phase, confirming that the deformed specimen (in this case a highly rejuvenated glass) is still fully glassy after severe plastic deformation. Scale bar, 20 nm.

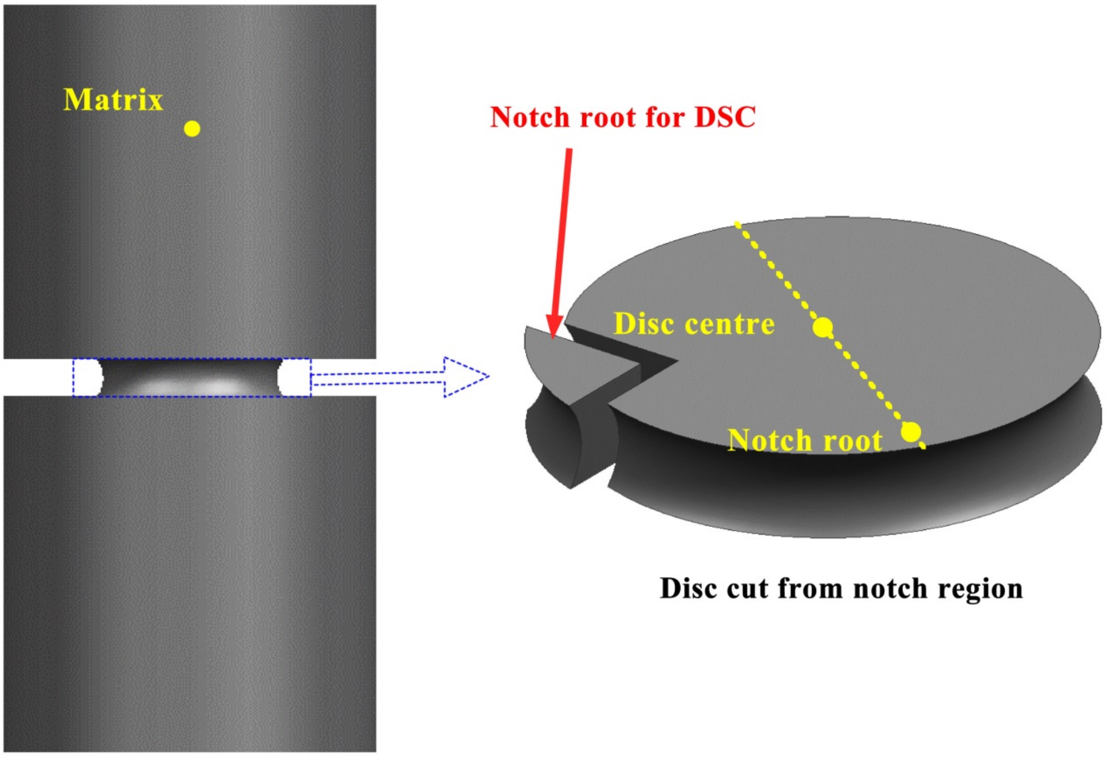

Supplementary Figure 3 │ Schematic illustration of a notched specimen and its various regions. When the specimen is loaded in compression, plastic flow extends through a cylindrical region of the sample centred on, but extending above and below, the disc defined by the circumferential notch. After deformation, this disc is cut from the specimen to allow measurements (differential scanning calorimetry, microhardness testing, and nanoindentation) of different regions. The matrix represents material far from the notch region. The measurements suggest that the material at the periphery of the disc (i.e. at notch root) is even more highly rejuvenated than at the disc centre.

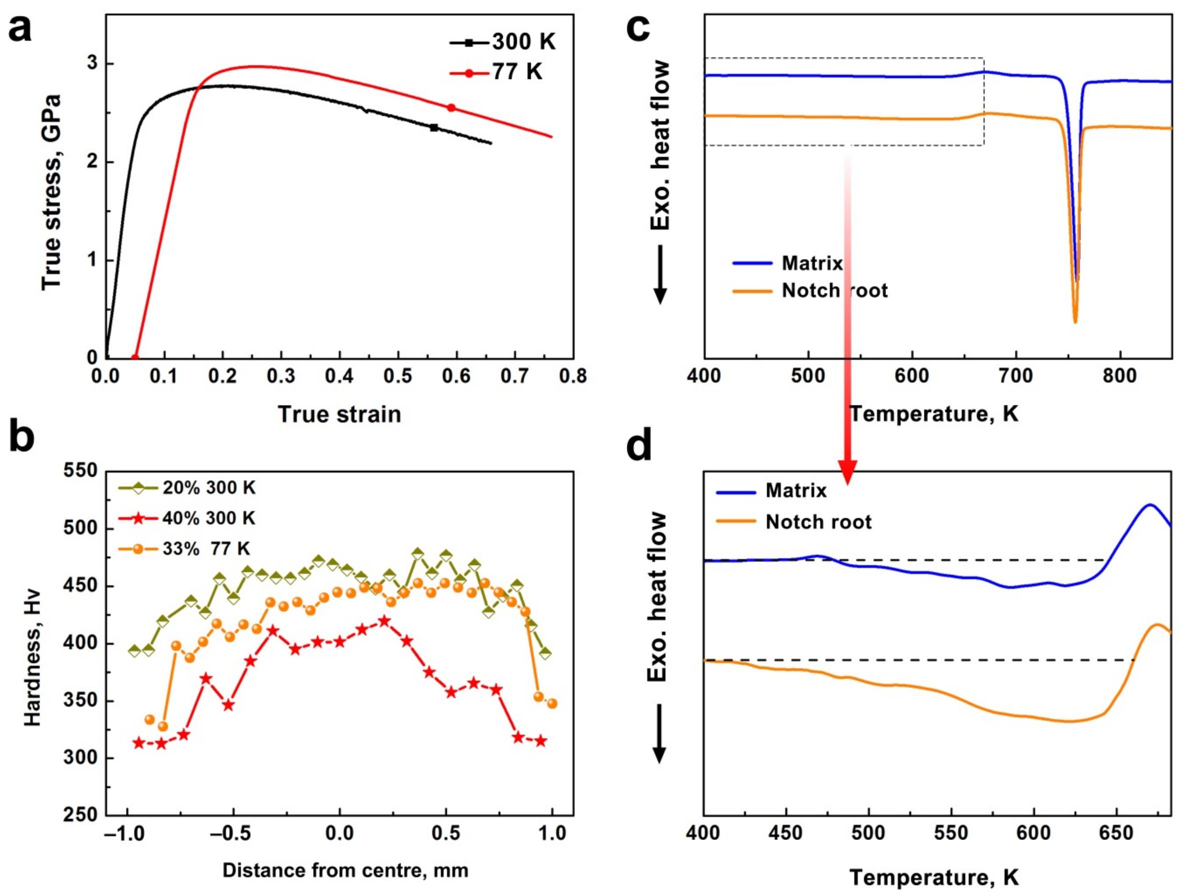

Supplementary Figure 4 │ Deformation-induced softening and rejuvenation in $Zr_{64.13}Cu_{15.75}Ni_{10.12}Al_{10}$ bulk metallic glass compressed at liquid-nitrogen temperature. **(a)** True stress-true strain curves of notched specimens under compression at RT or at liquid-nitrogen temperature (77 K); for clarity, the latter curve is displaced laterally. The yield stress at 77 K is slighter higher than that at RT; the applied axial stress reaches a maximum of 2.98 GPa, followed by a reduction. **(b)** Profiles of Vickers microhardness across the diameter of the disc (Supplementary Fig. 3) in specimens deformed at 300 K or 77 K. **(c,d)** DSC traces (heating rate 20 K min^{-1}) for samples taken from the matrix or from the notch root in the specimen subjected to 33% compressive strain at 77 K. For this specimen, the maximum reduction in hardness is ~33%, and the relaxation enthalpy ΔH_{rel} at the notch root is 2.74 kJ mol^{-1}; both these values are very similar to those measured or interpolated for specimens deformed to the same strain at RT. This suggests these that high levels of rejuvenation are close to saturation.

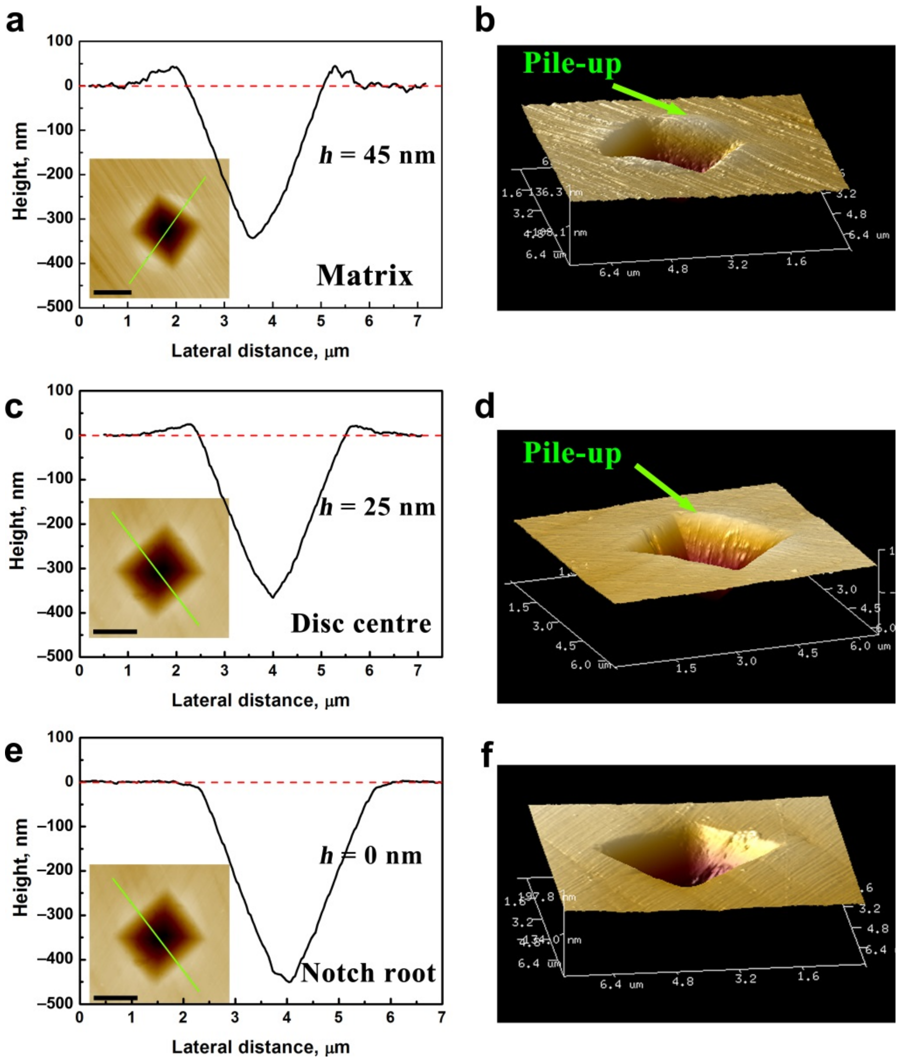

Supplementary Figure 5 | AFM line-scans and images of micro-indents in a BMG specimen subjected to 40% plastic strain at RT. The applied load on the micro-indenter is 5 g. The regions in the specimen are as in Supplementary Fig. 3: **(a,b)** the matrix (far from the notch region), **(c,d)** the centre of the disc defined by the peripheral notch, and **(e,f)** the perimeter of the disc (i.e. the notch root). A high level of rejuvenation is achieved in the disc, especially at the notch root. As the degree of rejuvenation increases, the pile-up height decreases: from 45 nm in the matrix (with obvious shear bands around the indent), to 25 nm at the disc centre, and disappearing at the notch root. As noted in Fig. 5, this trend to smaller pile-up is consistent with decreasing work-softening (yield drop) in the glass as its degree of prior deformation is increased. The scale bars in the insets of **a**, **c**, **e** are 2 μm.

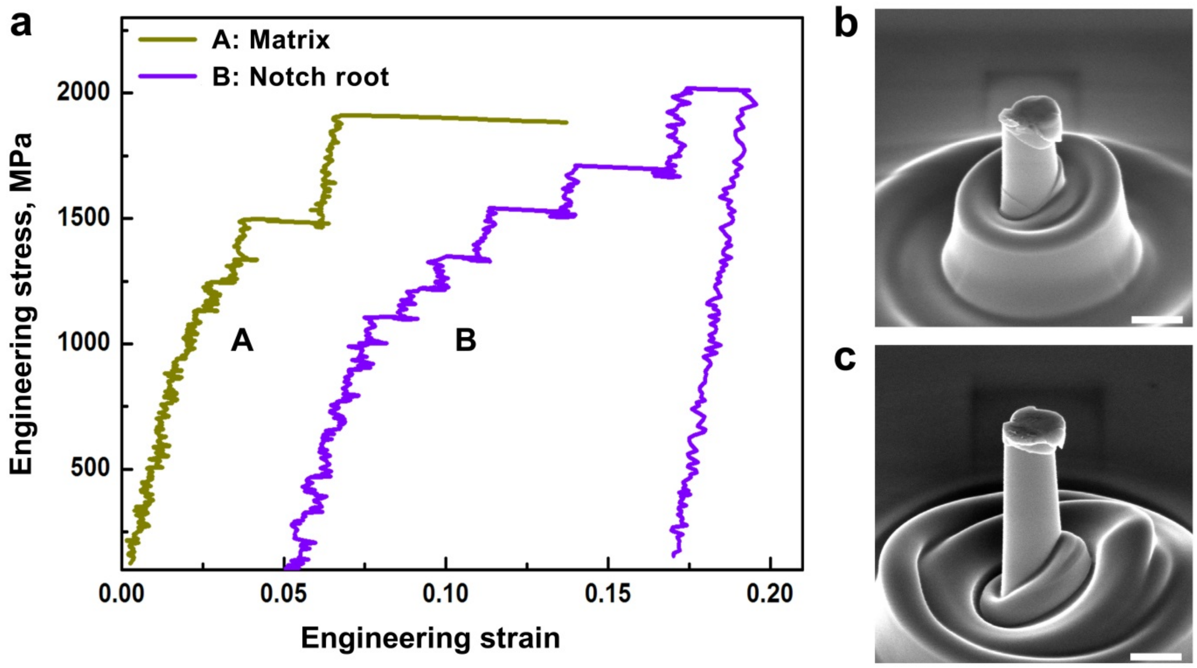

Supplementary Figure 6 │ Compressive behaviour of BMG pillars. The micro-pillars (~0.5 μm in diameter and 1.5–2.0 μm in height) were prepared by ion-milling using a low ion current of 100 pA (focused ion beam, FEI Scios) from a BMG specimen that had been subjected to 15% compressive strain. The pillars were loaded in compression using an Agilent G200 Nanoindenter under displacement control at a strain rate of 5×10^{-4} s^{-1}. The vertical taper of all pillars was controlled within 2° to ensure reliable interpretation of the mechanical test data. **(a)** Stress-strain curves for pillars extracted from the matrix (i.e. far from the notch region) and from the notch root (i.e. highly rejuvenated metallic glass). Both show discontinuous increases in strain (*pop-ins*). SEM images of the pillars **(b)** from the matrix and **(c)** from the notch root after extraction from the original notched specimen. Both show that the deformation process is governed by shear banding, consistent with the pop-ins seen in (a). There is no evidence of homogeneous deformation as reported in some earlier studies[2-4]. It is clear, however, that in the pillar extracted from the deformed notch region, the initial yielding is at lower stress, the transition from elastic to plastic is more gradual, and overall the pop-ins are more numerous and smaller, thus representing a trend towards more homogeneous deformation. The scale bars of **b** and **c** are 500 nm.

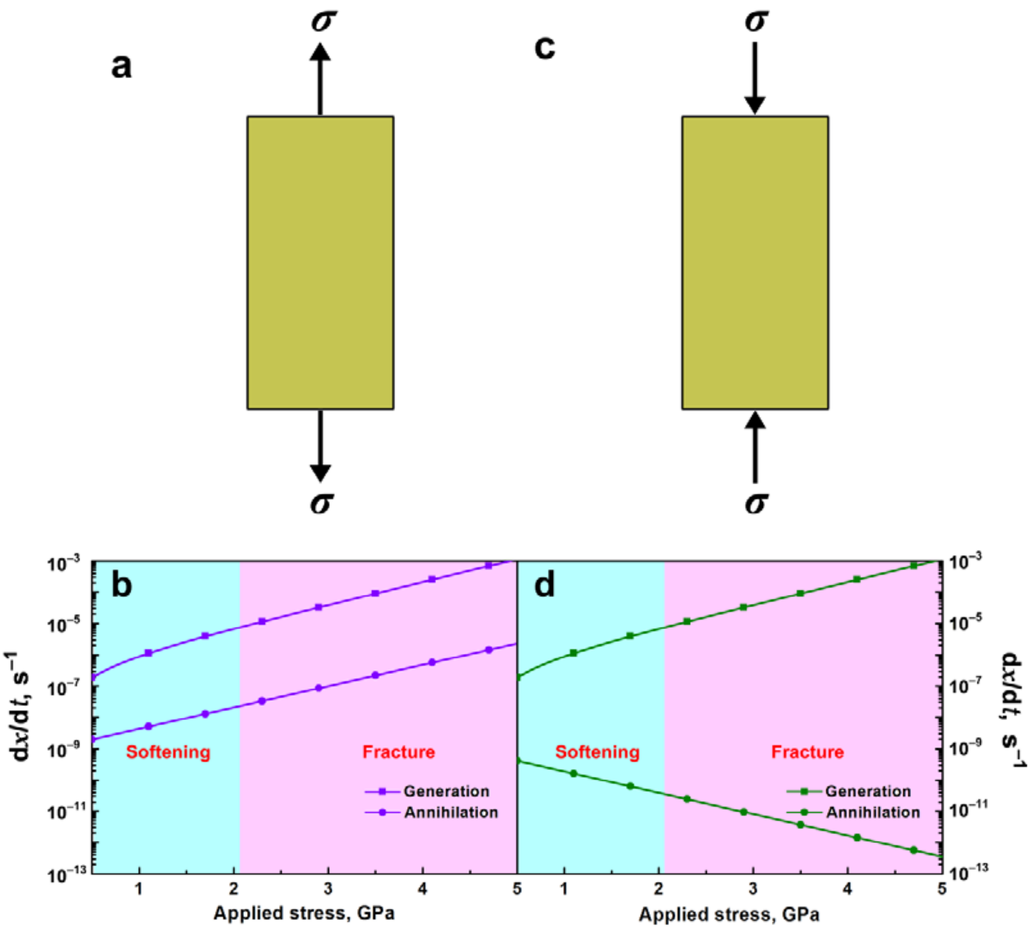

Supplementary Figure 7 │ Free-volume evolution in un-notched BMG specimens. (a,c) Schematic diagrams of specimens under tension and compression. (b,d) Free-volume generation and annihilation rates as a function of applied axial compressive or tensile stress. For a normal stress σ applied along the 1-axis, $\sigma_1 = \sigma$, $\sigma_2 = 0$, $\sigma_3 = 0$, $\sigma_m = (\sigma_1 + \sigma_2 + \sigma_3)/3 = \sigma/3$, and $\tau = \sigma/\sqrt{3} = 0.577\sigma$. The absolute values for mean stress σ_m and shear stress τ are the same for tension and compression, but the signs are opposite. The free-volume generation rates (Eq. (1)) are the same under tension and compression, but the annihilation rates (Eq. (2)) are very different. The net increases in free volume after integration of the rates as a function of stress at a given loading rate (10^{-3} GPa s^{-1}), are almost the same (Fig. 7b), because the generation rate is dominant and the annihilation rates are negligible in comparison. As shown in Fig. 7b, the net free volume increases rapidly at a critical stress (about 2 GPa); this leads to shear banding and catastrophic failure of the specimen rather than general rejuvenation.

The model underlying the plots in Fig. 7 and in **(b,d)** above is rather sensitive to the chosen input values of the parameters, specifically to the values of atomic volume Ω, shear modulus μ, initial reduced free volume x, activation volume V and Poisson's ratio ν. While changes in these parameters adjust the positions of the curves in Fig. 7 and in (b,d), the overall forms of free-volume evolution remain essentially unchanged.

Supplementary Table 1 │ Specimens studied in the present work. The specimens were loaded in uniaxial compression at a cross-head speed of 0.05 mm min^{-1}. The quoted values are for axial true plastic strain.

Samples	Processing
Un-notched sample	40% compressive strain at RT
Notched samples	20% compressive strain at RT
	30% compressive strain at RT
	40% compressive strain at RT
	40% compressive strain at RT + annealed at 573 K for 12 h
	33% compressive strain at 77 K

Supplementary References

1. Neuber, H. *Theory of Notch Stress*. (Edwards Brothers Inc., Ann Arbor, MI, 1946), p. 127.
2. Raghavan, R. et al. Ion irradiation enhances the mechanical performance of metallic glasses. *Scripta Mater*. **62,** 462–465 (2010).
3. Heo, J., Kim, S., Ryu, S., & Jang, D. Delocalized plastic flow in proton-irradiated monolithic metallic glasses. *Sci. Rep*. **6,** 23244 (2016).
4. Tönnies, D., Maaß, R. & Volkert, C. A. Room temperature homogeneous ductility of micrometer-sized metallic glass. *Adv. Mater.* **26,** 5715–5721 (2014).
